# Predictive Value of Comprehensive Geriatric Assessment Scores for Mortality in Patients With Hip Fracture: A Retrospective Cohort Study

**DOI:** 10.7759/cureus.45070

**Published:** 2023-09-11

**Authors:** Jehan Zaib, Abdulaziz Madni, Muhammad Saad Azhar

**Affiliations:** 1 Trauma and Orthopaedics, Hull University Teaching Hospitals, Hull, GBR; 2 Trauma and Orthopaedics, The Dudley Group NHS Foundation Trust, Birmingham, GBR

**Keywords:** american society of anesthesiologists classification, nottingham hip fracture score, clinical frailty score, scoring systems, mortality, hip fractures

## Abstract

Objective

To assess the predictive value of three scoring systems, namely the American Society of Anesthesiologists (ASA) classification, the Clinical Frailty Scale (CFS), and the Nottingham Hip Fracture Score (NHFS), in predicting mortality among patients with hip fractures.

Materials and methods

This retrospective cohort study included 628 participants aged 60 years and above who sought treatment at a UK hospital between January 2018 and December 2018. Data on age, gender, mortality, and assessment scores were collected. The area under the curve was calculated for each receiver operator characteristic (ROC). Cross-tabulation was performed to examine the association between various assessment scores and mortality using the chi-square test.

Results

The mean age was 80.80±11.18 years. Females were 408 (64.97%). Higher CFS (p<0.001) and NHFS (p<0.001) scores were significantly associated with mortality, while the ASA score did not show a significant association (p=0.225). The calculated area under the curve (AUC) values were as follows: 0.71 (95% CI: 0.65 to 0.76) for CFS, 0.46 (95% CI: 0.39 to 0.53) for NHFS, and 0.41 (95% CI: 0.34 to 0.48) for the ASA score. Utilizing a cut-off of ≥6 for CFS, 57 individuals (98.3%) in the 30-day mortality group were correctly identified. Similarly, the ROC analysis determined a ≥5 cut-off for NHFS accurately predicting 50 patients (86.2%) who deceased within 30 days. Applying an ASA ≥3 cut-off resulted in a predictive mortality rate of 56 (96.6%). The NHFS score demonstrated the highest predictive capability for mortality, with patients scoring ≥5 having a significantly higher risk of mortality compared to those with a score <5.

Conclusion

This study showed robust correlations between high CFS (≥6) and NHFS (≥5), and mortality within the hip fracture patient cohort.

## Introduction

The identification of elderly patients who are at high risk of experiencing unfavourable outcomes following hip fractures is of utmost importance due to the severe consequences associated with such injuries [1]. Given the significant annual incidence of over 60,000 hip fractures in the UK, the thoughtful allocation of healthcare resources is vital to maintain transparency and provide compassionate support to patients' families without raising false expectations [2]. Mortality rates linked to hip fractures can reach as high as 30% within one year, encompassing both early mortality within 30 days and delayed mortality at the one-year mark [3].

It is crucial for healthcare professionals to prioritize the use of these scoring systems to accurately identify high-risk patients and provide customized care, leading to improved outcomes and preventing unrealistic expectations that may inadvertently lead to unnecessary treatments [4]. The Nottingham Hip Fracture Score (NHFS) has shown consistent reliability in predicting 30-day mortality in hip fracture cases. Patients with a score below 4 are categorized as low-risk individuals. This scoring system integrates several independent predictors of mortality, including age, sex, medical conditions, mental health score, haemoglobin level, residency in an institution, and the presence of malignancy [5].

Frailty is a recognized medical condition that significantly impacts surgical and geriatric outcomes, as evidenced by several studies [6,7]. It refers to a state in which individuals with vulnerability have diminished physiological capacity to cope with external stressors such as trauma or infection. The Clinical Frailty Scale (CFS) is a practical and efficient bedside screening tool for assessing frailty among patients with hip fractures, ranging from level 1 (very fit) to level 9 (terminally ill) [8].

The American Society of Anesthesiologists (ASA) grading system is widely acknowledged as a valuable tool for evaluating the pre-anaesthesia medical co-morbidities of patients [9]. It plays a vital role in assessing perioperative risks and can be utilized in conjunction with other factors, including the type of surgery, frailty, and level of deconditioning, to determine the most suitable care approach. Research findings indicate that patients classified as ASA class 1-2 exhibit lower rates of rehospitalization and achieve better mental health scores at 6- and 12-month follow-ups compared to those classified as ASA 3. Moreover, these patients experience enhanced recovery of walking ability and overall health [10].

A study was conducted to evaluate the predictive capability of the CFS for 30-day and one-year mortality in older patients with proximal femur fractures. The study findings indicated that the CFS achieved a predictive accuracy of 69.9% in estimating mortality rates following proximal femur fractures [8].

The aim of this retrospective study was to assess the predictive value of three scoring systems, namely the ASA classification, CFS, and NHFS, in predicting mortality among patients with hip fractures.

## Materials and methods

The present retrospective observational study aimed to investigate the outcomes of 628 participants who sought treatment at the Department of Orthopaedics, Hull Royal Infirmary, United Kingdom. The study utilized electronic patient records that were available between January 1, 2018, and December 31, 2018. Subsequently, the participants were followed for a duration of two years until December 31, 2020. To ensure the protection of patient confidentiality, stringent measures were implemented throughout the study, and the collected data was exclusively used for research purposes. Prior to the initiation of data collection, the study protocol underwent registration with the hospital's audit committee, adhering to established ethical and consensual guidelines. The Hull University Teaching Hospital Audit Team issued approval number 2019-210.

The study encompassed all hip fractures occurring in individuals aged 60 years and above, including both intracapsular and extracapsular neck of femur fractures. A follow-up period of two years was implemented to assess mortality rates at both the one-year and two-year marks. Furthermore, comorbidity association data was collected specifically for patients who experienced mortality within the first year. Age group (years), gender and death status were recorded. To obtain comprehensive patient information, various parameters were recorded at the time of admission. This included the assessment of the ASA classification [11], CFS [12], and NHFS [13] for each individual patient.

The collected data were entered into Microsoft Excel sheet 2016 (Microsoft Corporation, Redmond, USA) and imported into R programming 4.1.2 (R Foundation for Statistical Computing, Vienna, Austria) for analysis. Descriptive statistics, including means, standard deviations, and percentages, were employed to summarize the data. Receiver Operating Characteristic (ROC) curves were constructed to evaluate the predictive capability of the CFS, NHFS, and ASA score for mortality. The Area Under the Curve (AUC) was calculated for each ROC. Cross-tabulation was performed to examine the association between various assessment scores and mortality using the chi-square test. The predictive ability of the assessment scores for mortality was stratified at different time points. Logistic regression analysis was conducted with mortality as the dependent variable and the assessment scores as independent variables. A significance level of p<0.05 was considered statistically significant.

## Results

The mean age of the participants was 80.80±11.18 years. Table 1 displays the distribution of age, gender, death status, time of mortality, and mortality assessment scores within the study population. Regarding age, the majority of individuals were aged 71 and above, accounting for 537 individuals (85.51%). The age groups of 51-70, 31-50, and 17-30 constituted 80 (12.74%), 9 (1.43%), and 2 (0.32%) individuals, respectively. In terms of gender, 408 individuals (64.97%) were female, while 220 individuals (35.03%) were male. Among the study population, 416 individuals (66.24%) were reported as alive, whereas 212 individuals (33.76%) were reported as deceased. Mortality assessment scores were evaluated using the CFS, ASA, and NHFS scores. For the CFS score, 215 individuals (34.24%) had scores less than 6, while 413 individuals (65.76%) had scores of 6 or higher. Concerning the ASA score, 16 individuals (2.55%) scored below 3, whereas 612 individuals (97.45%) scored 3 or above. Regarding the NHFS score, 112 individuals (17.83%) obtained scores lower than 5, and 516 individuals (82.17%) achieved scores of 5 or greater.

**Table 1:** Distribution of age, gender, death status, time of mortality, and mortality assessment scores ASA: American Society of Anesthesiologists classification; CFS: Clinical Frailty Scale; NHFS: Nottingham Hip Fracture Score

Variable	Characteristic	n(%)
Age group (years)	17-30	2 (0.32)
	31-50	9 (1.43)
	51-70	80 (12.74)
	71 & above	537 (85.51)
Gender	female	408 (64.97)
	male	220 (35.03)
Status	Alive	416 (66.24)
	Dead	212 (33.76)
CFS score	<6	215 (34.24)
	≥6	413 (65.76)
ASA score	<3	16 (2.55)
	≥3	612 (97.45)
NHFS score	<5	112 (17.83)
	≥5	516 (82.17)

**Figure 1:**
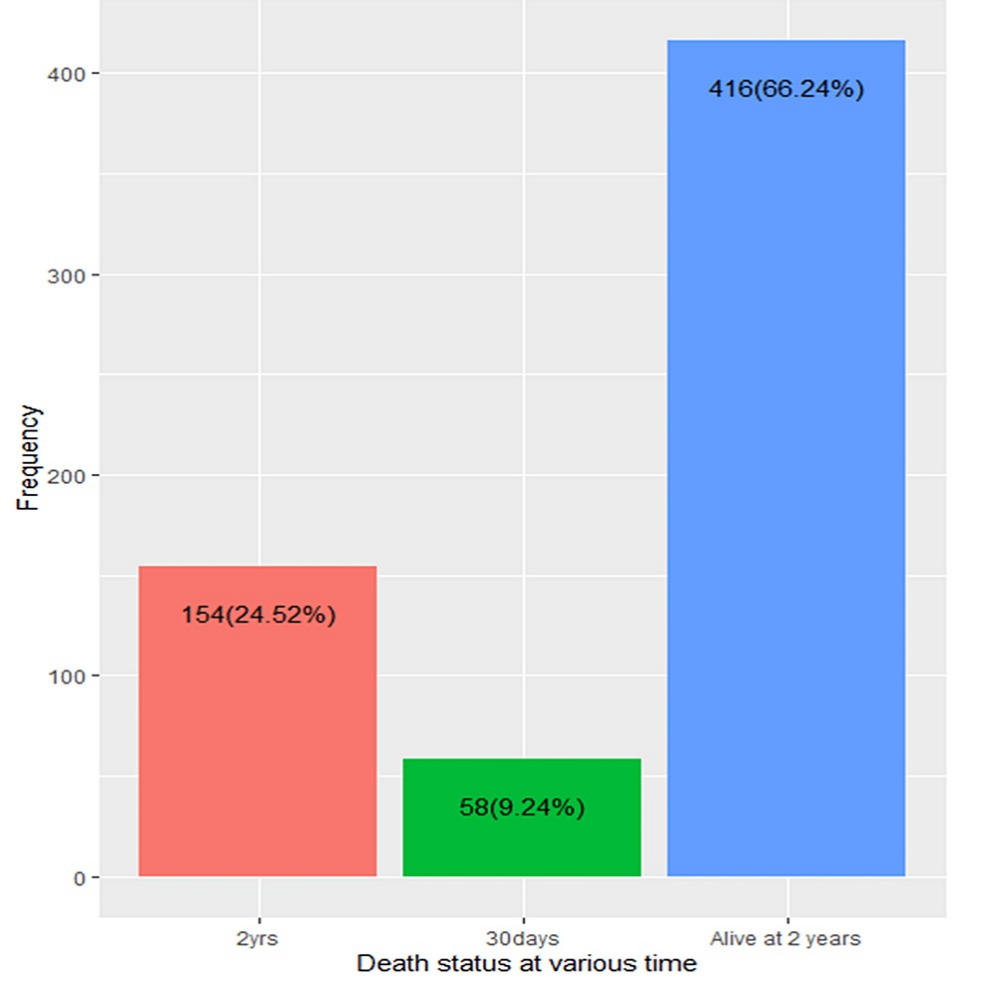
Death status of the participants

Within the study population, 154 individuals (24.52%) experienced mortality within 2 years, and 58 individuals (9.24%) within 30 days. Additionally, 416 individuals (66.24%) were still alive at the 2-year mark (Figure 1).

Table 2 compares different scores for the assessment of mortality, namely the CFS score, NHFS, and ASA score. For the ASA score, among individuals with a CFS score less than 6, seven (3.26%) had an ASA score less than 3, while nine (2.18%) had an ASA score of 3 or higher. Similarly, among individuals with a CFS score of 6 or higher, 208 (96.74%) had an ASA score of 3 or higher, while 404 (97.82%) had an ASA score less than 3. There was no significant difference between CFS score and ASA score (p=0.585). Regarding the NHFS score, among individuals with a CFS score less than 6, 28 (13.02%) had an NHFS score less than 5, while 84 (20.34%) had an NHFS score of 5 or higher. Among individuals with a CFS score of 6 or higher, 187 (86.98%) had an NHFS score of 5 or higher, while 329 (79.66%) had an NHFS score less than 5. There was a statistically significant difference between CFS score and NHFS score (p=0.031).

**Table 2:** Comparison of various scores for assessment of mortality *Chi-square test ASA: American Society of Anesthesiologists classification; CFS: Clinical Frailty Scale; NHFS: Nottingham Hip Fracture Score

Variable	Characteristic	CFS		p-value^*^
		<6	≥6	
ASA Score	<3	7 (3.26)	9 (2.18)	0.585
	≥3	208 (96.74)	404 (97.82)	
NHFS	<5	28 (13.02)	84 (20.34)	0.031
	≥5	187 (86.98)	329 (79.66)	

Among deceased individuals, 210 (50.48%) had a CFS score less than 6, whereas a substantial majority of 203 (95.75%) had a CFS score of 6 or higher. A highly significant association exists between CFS score and mortality (p<0.001). For ASA score, 403 (96.88%) had an ASA score less than 3, whereas 209 (98.58%) had an ASA score of 3 or higher. However, the association was not statistically significant between ASA score and mortality (p=0.309). For the NHFS score, among deceased individuals, 358 (86.06%) had an NHFS score less than 5, whereas 158 (74.53%) had an NHFS score of 5 or higher. The association between NHFS score and mortality was significant (p<0.001) (Table 3).

**Table 3:** Predictive ability of various scores used for assessment of mortality *Chi-square test ASA: American Society of Anesthesiologists classification; CFS: Clinical Frailty Score; NHFS: Nottingham Hip Fracture Score

Variable	Characteristic	Mortality		p-value^*^
		Alive (n = 416)	Dead, (n = 212)	
CFS	<6	206 (49.52)	9 (4.25)	<0.001
	≥6	210 (50.48)	203 (95.75)	
ASA Score	<3	13 (3.12)	3 (1.42)	0.309
	≥3	403 (96.88)	209 (98.58)	
NHFS	<5	58 (13.94)	54 (25.47)	<0.001
	≥5	358 (86.06)	158 (74.53)	

Table 4 presents the predictive ability of various scores used for the assessment of mortality at different time points. For the CFS score, among individuals assessed at 2 years, eight (5.19%) with a CFS score less than 6 experienced mortality, while only one (1.72%) with a CFS score of 6 or higher died. Among individuals assessed at 30 days, 146 (94.81%) with a CFS score less than 6 survived, while 57 (98.28%) with a CFS score of 6 or higher were still alive. There was a highly significant association between CFS score and mortality at both time points and overall survival (p<0.01). Regarding the ASA score, among individuals assessed at 2 years, only one (0.65%) with an ASA score less than 3 experienced mortality, while two (3.45%) with an ASA score of 3 or higher died. Among individuals assessed at 30 days, 153 (99.35%) with an ASA score less than 3 survived, and 56 (96.55%) with an ASA score of 3 or higher were still alive. However, the association was not significant (p=0.225). For the NHFS score, among individuals assessed at 2 years, 47 (30.52%) with an NHFS score less than 5 experienced mortality, while seven (12.07%) with an NHFS score of 5 or higher died. Among individuals assessed at 30 days, 107 (69.48%) with an NHFS score less than 5 survived, and 51 (87.93%) with an NHFS score of 5 or higher were still alive. The significant association between NHFS score and mortality at both time points and overall survival was highly significant (p<0.001).

**Table 4:** Predictive ability of various scores used for assessment of mortality at various time points ASA: American Society of Anesthesiologists classification; CFS: Clinical Frailty Scale; NHFS: Nottingham Hip Fracture Score

Variable	Characteristic	Mortality at various time			p-value^*^
		2yrs, n= 154	30days, n= 58	Alive, n= 416	
CFS	<6	8 (5.19)	1 (1.72)	206 (49.52)	<0.001
	≥6	146 (94.81)	57 (98.28)	210 (50.48)	
ASA score	<3	1 (0.65)	2 (3.45)	13 (3.12)	0.225
	≥3	153 (99.35)	56 (96.55)	403 (96.88)	
NHFS	<5	47 (30.52)	7 (12.07)	58 (13.94)	<0.001
	≥5	107 (69.48)	51 (87.93)	358 (86.06)	

For the NHFS, patients with a score <5 had a mortality rate of 87.0% at 2 years and 13.0% at 30 days. In the multivariable analysis, patients with a score ≥5 had a significantly higher risk of mortality compared to those with a score <5 (OR=3.20, 95% CI 1.43-8.19, p=0.008) at 2 years. This association remained significant after adjusting for relevant covariates (multivariable OR=3.24, 95% CI 1.44-8.30, p=0.008). Regarding the CFS, patients with a score <6 had a mortality rate of 88.9% at 2 years and 11.1% at 30 days. However, in the multivariable analysis (Table 5), there was no significant association between a CFS score ≥6 and mortality at 2 years (OR=3.12, 95% CI 0.56-58.59, p=0.288) or 30 days (OR=3.32, 95% CI 0.58-62.66, p=0.267).

**Table 5:** Multivariate analysis for NHFS and CFS CFS: Clinical Frailty Scale; NHFS: Nottingham Hip Fracture Score

Assessment tool	Characteristics	Time of mortality		OR (univariable)	OR (multivariable)
		2yrs mortality	30days mortality		
NHFS	<5	47 (87.0)	7 (13.0)	-	-
	≥5	107 (67.7)	51 (32.3)	3.20 (1.43-8.19, p=0.008)	3.24 (1.44-8.30, p=0.008)
CFS	<6	8 (88.9)	1 (11.1)	-	-
	≥6	146 (71.9)	57 (28.1)	3.12 (0.56-58.59, p=0.288)	3.32 (0.58-62.66, p=0.267)

Table 6 presents the comorbid conditions of patients who experienced hip fractures and subsequently died within 30 days. Among the comorbidities observed, dementia had the highest prevalence, with 36 patients accounting for 62.1% of the cases. Other prevalent comorbidities included hypertensive conditions (36.21%), diabetes mellitus (24.14%), and ischemic heart disease (31.03%). Additionally, conditions such as atrial fibrillation (22.41%), chronic kidney disease (24.14%), and active cancer (12.07%) were also observed in a considerable number of patients who experienced hip fracture and subsequently died within 30 days.

**Table 6:** Comorbid conditions of patients with a hip fracture who died within 30 days

Comorbidities	n(%)
Blind	6 (10.34)
Deaf	1 (1.72)
Hypertensive	21 (36.21)
Diabetes Mellitus	14 (24.14)
Asthma	4 (6.9)
Chronic Obstructive Pulmonary Disease	6 (10.34)
Angina	3 (5.17)
Ischemic Heart Disease	18 (31.03)
Atrial Fibrillation	13 (22.41)
Cerebral Vascular Accident	11 (18.97)
Thyroid Disorder	4 (6.9)
Active Cancer	7 (12.07)
Past Cancer	4 (6.9)
Chronic Kidney Disease	14 (24.14)
Acute Kidney Injury	3 (5.17)
Infection	3 (5.17)
Dialysis	4 (6.9)
Deep Vein Thrombosis	1 (1.72)
Dementia	36 (62.1)

**Figure 2:**
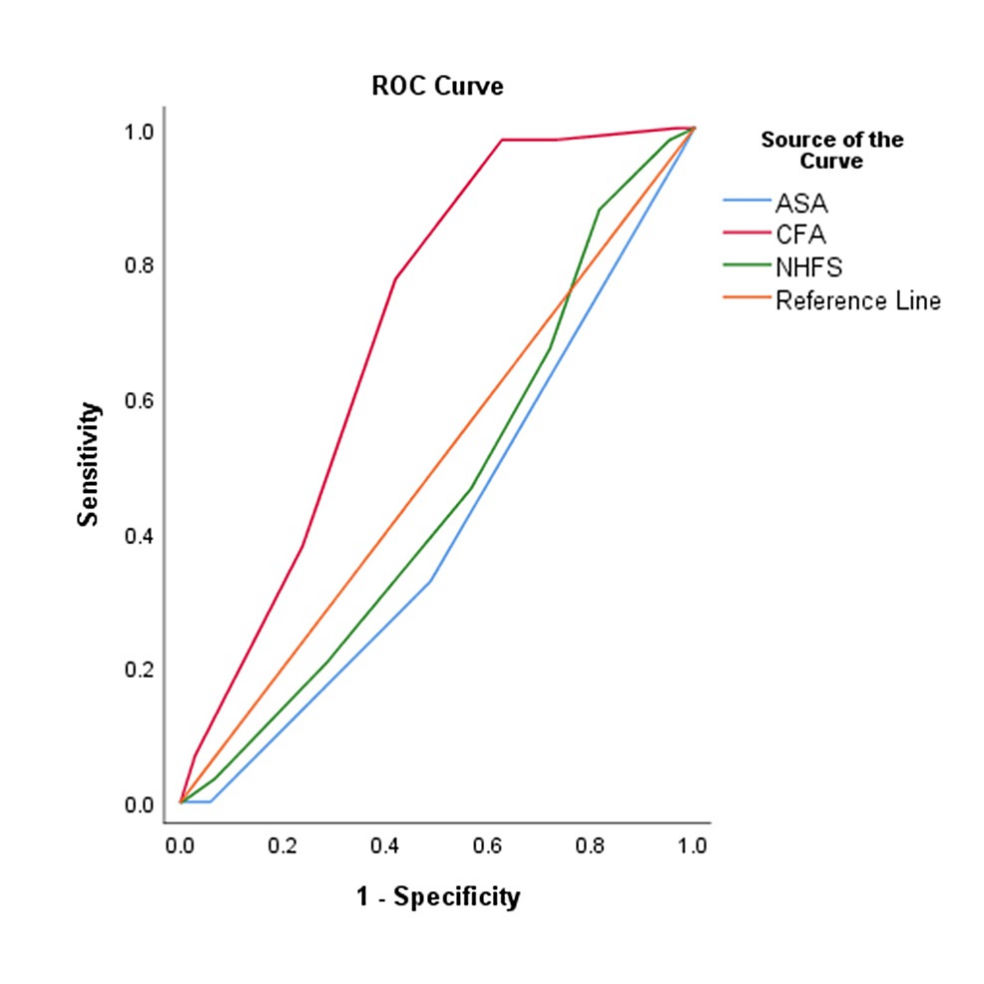
ROC for CFS, NHFS and ASA scores for prediction of mortality ASA: American Society of Anesthesiologists classification; CFS: Clinical Frailty Scale; NHFS: Nottingham Hip Fracture Score; ROC: receiver operator characteristic

The area under the curve (AUC) values were calculated as 0.71 (95% CI: 0.65 to 0.76) for CFS, 0.46 (95% CI: 0.39 to 0.53) for NHFS, and 0.41 (95% CI: 0.34 to 0.48) for ASA. Figure 2 presents the receiver operating characteristic (ROC) curve for comparing the three scores. For the CFS, a cut-off of >=6 was identified. Among the patients in the 30-day mortality group, 57 (98.3%) individuals were found to have died when this predictive cut-off was applied. Similarly, the NHFS cut-off of >=5 was determined from the ROC analysis, and with this criteria, 50 (86.2%) of the patients who died within 30 days were accurately predicted. The ASA cut-off of >=3 resulted in a predictive mortality rate of 56 (96.6%).

## Discussion

This study aimed to assess the predictive capacity of three scoring systems (ASA Classification, Clinical Frailty Score, and Nottingham Hip Fracture Score) for mortality in hip fracture patients. We found that dementia was the most prevalent comorbidity among patients who died within 30 days after hip fracture. CFS and NHFS scores were associated with mortality, while ASA scores were not. NHFS scores of 5 or higher increased the risk of mortality. CFS had the highest predictive ability for mortality.

The average age of participants with hip fractures in the study was 80.80±11.18 years. The majority of participants (85.51%) were aged 71 and above. Older individuals, especially those over 71, are more prone to hip fractures due to age-related factors, decreased bone strength, increased fall risk, comorbidities, and reduced recovery ability [14]. Previous studies also reported similar findings that most of the patients suffering from hip fractures were above 70 years [15, 16].

In our study, there were more females with hip fractures than males. This could be attributed to factors such as the higher prevalence of osteoporosis in women, hormonal changes during menopause, differences in bone structure, increased risk of falls, and potential differences in healthcare-seeking behaviour [17]. A study conducted in Argentina reported that females were more affected by hip fractures than males (female/male ratio: 2.96) [18].

According to our findings, a significant percentage of participants experienced mortality within specific time frames. Specifically, 24.52% of the participants passed away within 2 years, while 9.24% experienced mortality within 30 days. However, the majority of participants (66.24%) were still alive at the 2-year mark. A previous study revealed a 1-year postoperative mortality rate of 27.3%. Furthermore, by the end of the follow-up period, the mortality rate after hip fracture reached 79.0% [19].

Our results revealed that the most prevalent comorbidity among patients with hip fracture who died within 30 days after hip fracture was dementia, followed by hypertensive conditions, diabetes mellitus, and ischemic heart disease. The high prevalence of comorbidity among patients with hip fractures is due to shared risk factors with several chronic diseases, such as diabetes mellitus, chronic pulmonary disease, dementia, and cancer. Osteoporosis and hip fractures have overlapping risk factors with these conditions, which explains why the prevalence of comorbidity is higher in hip fracture patients compared to the general population [20].

The current study found a significant association between CFS and NHFS with mortality, meaning that these two factors were found to have a notable impact on the likelihood of mortality. On the other hand, the study did not find a significant association between the ASA score and mortality. A study involving 509 patients aimed to assess the effectiveness of the CFS in predicting mortality rates within 30 days and one year following a hip fracture in older patients. The findings of the study indicated that higher CFS scores were linked to increased mortality [8]. A separate study, involving 666 patients, found that a higher NHFS was associated with an elevated risk of mortality [21].

The area under the curve (AUC) values for CFS, NHFS, and ASA were calculated as 0.71, 0.46, and 0.41, respectively. CFS had the highest AUC value, indicating better predictive ability for mortality. In a previous study [8], it was shown that the CFS had a higher discriminative ability in predicting mortality compared to the ASA score and the patient's age. The area under the curve (AUC) for the CFS was 0.699 (95% CI 0.651 to 0.747), while the AUC for the ASA score was 0.634 (95% CI 0.576 to 0.691) and for the patient's age was 0.585 (95% CI 0.523 to 0.648).

Limitations

The study presented notable insights into the predictive capacities of different scoring systems for mortality in hip fracture patients. However, several limitations warrant consideration in interpreting its results. The study's retrospective design, reliant on historical medical records, introduces potential inaccuracies and missing data. While the study acknowledged comorbidities, it did not explore their potential impacts on scoring system outcomes through meaningful group stratification. Similarly, the study did not delve into the potential influence of patients' mobility statuses, a crucial factor affecting mortality rates, especially given its inclusion in the NHFS and CFS but not in the ASA score. Moreover, relying on a single centre and not accounting for the influence of ethnicity and culture might introduce biases that restrict the generalizability of the scoring systems' effectiveness. To address these limitations and enhance the validity of the findings, a prospective multicenter study could be instrumental. Such a study would provide more robust data collection, encompass diverse patient populations, and enable the examination of potential variations in scoring system performance across different healthcare settings and demographic groups.

## Conclusions

The study revealed that both the Clinical Frailty Scale (CFS) >=6 and Nottingham Hip Fracture Score (NHFS) >=5 showed strong associations with mortality among patients with hip fractures. Conversely, the American Society of Anesthesiologists (ASA) score did not demonstrate a significant association with mortality.
